# *NTRK2* Promotes Sheep Granulosa Cells Proliferation and Reproductive Hormone Secretion and Activates the PI3K/AKT Pathway

**DOI:** 10.3390/ani14101465

**Published:** 2024-05-14

**Authors:** Yuhang Jia, Yufang Liu, Peng Wang, Ziyi Liu, Runan Zhang, Mingxing Chu, Ayong Zhao

**Affiliations:** 1 College of Animal Science and Technology, College of Veterinary Medicine, Zhejiang Agriculture and Forestry University, Hangzhou 311300, China; 15645551808@163.com; 2 State Key Laboratory of Animal Biotech Breeding, Institute of Animal Science, Chinese Academy of Agricultural Sciences (CAAS), Beijing 100193, China; aigaiy@126.com (Y.L.); wp05223414@163.com (P.W.); zeeyyyyi@163.com (Z.L.); zhangrunan818@163.com (R.Z.)

**Keywords:** sheep, granulosa cells proliferation, steroid hormone secretion, *NTRK2*, PI3K/AKT pathway

## Abstract

**Simple Summary:**

Neurotrophin receptor B (*NTRK2*) is a member of the neurotrophic tyrosine receptor kinase (*NTRK*) family, and recent studies have shown that the *NTRK2* gene plays an important role in mammalian reproduction. The expression of the *NTRK2* gene is often closely related to the production and quality of gametes in mammals. In the present study, we found that overexpression of the *NTRK2* gene upregulated the expression of genes associated with cell proliferation and genes associated with steroid synthesis in sheep granulosa cells, and then verified this result by examining cell proliferation levels and hormone levels. We further demonstrated that *NTRK2* activates the PI3K-AKT signaling pathway, which is well known to regulate cell proliferation. These findings suggest that *NTRK2* plays a role in regulating the proliferation and hormone secretion of sheep GCs through the PI3K-AKT pathway. These studies provide valuable insights into the regulatory role of the *NTRK2* gene in follicular development in sheep.

**Abstract:**

Neurotrophin receptor B (*NTRK2*), also named *TRKB*, belongs to the neurotrophic factor family. Previous studies have shown that *NTRK2* is associated with high fertility in mammals. However, the molecular mechanism and regulatory pathway of this neurotrophic factor remain unclear. In this study, *NTRK2* overexpression and *NTRK2*-siRNA were constructed to detect the effects of *NTRK2* on the proliferation and hormone secretion of the ovarian granulosa cells (GCs) of sheep. We successfully isolated follicular phase granulosa cells in vitro from the ovaries of sheep in simultaneous estrus, and the immunofluorescence results confirmed that *NTRK2* was expressed in the collected cells. Subsequently, the effect of *NTRK2* on the proliferation of sheep granulosa cells was examined via cell transfection experiments. The results showed that the expression of *CDK4* and *CyclinD2* was significantly increased after *NTRK2* overexpression, while the opposite trend was observed after the inhibition of *NTRK2* expression (*p* < 0.05). The EdU and CCK-8 assays showed that the proliferation rate of sheep GCs was significantly increased after *NTRK2* overexpression, while the opposite trend was observed after the inhibition of *NTRK2* expression (*p* < 0.05). Moreover, *NTRK2* significantly increased the expression of steroidogenesis-related genes, including steroidogenic acute regulatory protein (*STAR*) and hydroxy-δ-5-steroid dehydrogenase (*HSD3B1*), and cytochrome P450 family 19 subfamily A member 1 (*CYP19A1*). The ELISA results showed that the secretion levels of E_2_ and P_4_ significantly increased after *NTRK2* overexpression, while the opposite trend was observed after the inhibition of *NTRK2* expression (*p* < 0.05). Previous studies had confirmed that *NTRK2* gene belongs to the PI3K-AKT signaling pathway and participates in the signaling of this pathway. This was demonstrated by protein–protein interaction analysis and NTRK2 belongs to the PI3K-AKT pathway. The modification of PI3K and AKT, markers of the PI3K-AKT pathway, via phosphorylation was increased after *NTRK2* overexpression in the sheep GCs, while the opposite trend was observed after the inhibition of *NTRK2* expression (*p* < 0.05). Overall, these results suggest that the *NTRK2* gene regulates the proliferation of GCs and the secretion of steroid hormones in sheep, and that it influences the phosphorylation level of the PI3K/AKT signaling pathway. These findings provided a theoretical basis and new perspectives for exploring the regulation of *NTRK2* gene in the development of ovine follicles.

## 1. Introduction

Developmental follicle formation requires close interaction between granulosa cells (GCS) and oocytes. In response to various hormones and signaling molecules (e.g., gonadotropins), granulosa cells proliferate, differentiate, and exchange substances and signals with surrounding granulosa cells, oocytes, and cumulus cells through gap junctions [1,2,3,4]. In turn, oocytes can also secrete various factors to interact with granulosa cells. Numerous studies have shown that the proliferation of GCs is associated with follicle development and ovarian ovulation [5,6,7]. However, our overall knowledge of the biological functions of granulosa cell growth and development and how molecular regulatory mechanisms affect oocyte maturation and follicular development is incomplete, and additional factors need to be explored to fully understand how granulosa cells contribute to follicular development in different species. Neurotrophic receptor tyrosine kinase 2 (*NTRK2*) is a member of the neurotrophic factor–tyrosine receptor kinase (*NTRK*) family, and other members of the family include neurotrophic receptor tyrosine kinase 1 (*NTRK1*) and neurotrophic receptor tyrosine kinase 3 (*NTRK3*), which function via the activation of their corresponding ligands [8,9,10,11,12]. Initial studies focused on the role of these proteins in neuron development in the nervous system, including their influence on growth [13], development [14], differentiation, and maintenance [15]. As research advanced, it was found that both the neurotrophic factor family and its receptors were expressed in these cells and mediated blastocyst formation and oocyte development through the activation of downstream pathways in non-neurological studies related to ovarian development and gamete formation [16,17].

Esmaeili-Fard et al. have identified *NTRK2* as a novel candidate gene for litter size in Baluchi sheep through genome-wide association studies and pathway analysis, which prompted us to investigate its function in ovarian granulosa cells in sheep [18]. Thus far, *NTRK2* has been identified in humans [19], mice [17], chickens [20], zebrafish [21,22], and other species, and its expressions and functions have been reported to varying degrees. *NTRK2* plays a key role in early follicle development in mice [23]. Compared to wild-type rats, female *NTRK2*-knockout (KO) rats have significantly reduced numbers of primary and secondary preovulatory follicles and exhibit marked retardation of follicle development and significant defects in follicle formation and development; moreover, when the *NTRK2* inhibitor K-252a is added to the in vitro oocyte maturation medium, oocyte maturation and blastocyst rates are significantly decreased, and the number of apoptotic blastocysts is significantly increased [24]. During chicken ovulation, *NTRK2* mRNA is localized in the granulosa cells layers of the secondary and preovulatory follicles of domestic chickens prior to ovulation, and *NTRK2* mRNA expression increases with follicle development [20]. In cattle, brain-derived neurotrophic factor (*BDNF)* promotes the proliferation of bovine GCs in follicles through *NTRK2*-mediated proliferative signaling [25]. These results suggest that *NTRK2* contributes to oocyte maturation and reduces apoptosis in blastocysts. Therefore, *NTRK2* and its related pathways may be important regulators of embryonic development, oocyte maturation, and related complications.

Although neurotrophic factors and their receptors are expressed in different tissues of animal species, fewer studies provide insight into the molecular mechanisms by which *NTRK2* regulates reproductive traits in sheep. It has been found that members of the neurotrophin family can bind to the high-affinity receptor tyrosine kinase A (*TrkA*) and the low-affinity receptor *p75* to activate different signaling pathways in oocytes to influence their maturation and ovulation [26]. *TrkA* receptors mainly activate phosphatidylinositol-3 kinase (*PI3K*) and mitotic-activated protein kinase (*MAPK*) to promote cells’ survival and proliferation [27]. In previous studies, neurotrophic factors and their receptors have been shown to play facilitative roles in ovarian development, oocyte maturation, and blastocyst formation in animals [28,29,30,31,32,33]. PI3K/AKT signaling is an important pathway that affects mammalian reproduction. Protein phosphorylation is one of the most common and important post-translational modifications, and it plays an important role in gene transcription and expression, cell proliferation, differentiation, apoptosis, and signal transduction in organisms [34]. We hypothesized that *NTRK2* affects the function of ovarian granulosa cells and, thus, follicle development and oocyte maturation in sheep through the PI3K/ATK signaling pathway. The results of this study provide new insights for in-depth study of the biological functions of *NTRK2* and the molecular regulatory mechanisms of *NTRK2* in regulating GC proliferation.

## 2. Materials and Methods

### 2.1. Isolation and Culture of Sheep Granulosa Cells In Vitro

The ovaries of 20 small-tailed Han sheep collected in this experiment came from a slaughterhouse near Langfang City, Hebei Province. The sheep that underwent ovary collection were identical in weight, age, and breeding environment. Sheep were subjected to simultaneous estrus for 12 days using an internal controlled release supposant (CIDR, progesterone 300 mg, Inter Ag Co., Ltd., Hamilton, New Zealand). Then, 45–48 h after CIDR removal (i.e., follicular phase, FP), the sheep were euthanized and ovarian tissue was immediately collected and brought back to the laboratory for granulosa cell collection. After disinfection with 75% alcohol, the samples were washed three times with pre-warmed saline containing 2% penicillin–streptomycin solution (SP, Gibco, Grand Island, NE, USA). After that, 1 mL of pre-warmed DMEM medium containing 10% fetal bovine serum (DMEM, Gibco, Grand Island, NE, USA) was aspirated with a 5 mL syringe, and follicular fluid was extracted from healthy follicles that were 2–6 mm in diameter and the follicular walls were scraped. The GCs were precipitated by centrifugation at 1500 rpm for 5 min and the supernatant was removed; this procedure was repeated twice. The cells were inoculated into 10 cm culture dishes (Corning, NY, USA) and cultured in a CO_2_ incubator (37 °C, 5% CO_2_, saturated humidity). When the density of the granulosa cell reached 70%, the cells were inoculated into 6-well plates, and immunofluorescence identification of the isolated cells was performed using the granulosa cell-specific antibody FSHR [35].

### 2.2. Granulosa Cell Immunofluorescence Staining

Ovarian granulosa cells were cultured at a density of 1 × 10^6^ per well in 6-well plates fitted with a round coverslip. The granulosa cells were fixed with 4% paraformaldehyde (Solarbio Science & Technology Co., Ltd., Beijing, China) for 30 min and washed 3 times with PBS (Gibco, Grand Island, NE, USA) for 5 min each time. The cells were permeabilized with 0.1% TritonX-100 (Solarbio Science & Technology Co., Ltd., Beijing, China) at 4 °C for 15 min, washed 3 times with PBS for 5 min, and then blocked with 10% goat serum (Solarbio Science & Technology Co., Ltd., Beijing, China) for 30 min. After that, anti-NTRK2 was added, and the cells were incubated overnight at 4 °C. Next, the cells were washed 3 times with PBS for 5 min each time. The cells were incubated with the appropriate amount of fluorescent IgG at 37 °C for 1 h and washed 3 times with PBS for 5 min each time. The nuclei were stained with DAPI (Solarbio Science & Technology Co., Ltd., Beijing, China) for 5 min in the dark, and then the cells were rinsed with PBS 5 times for 5 min each time. After treatment with a fluorescence quenching agent (Thermo Fisher, Waltham, MA, USA), the slides were sealed with resin glue and observed under a fluorescence inverted microscope (Leica, Wetzlar, Germany) to collect images.

### 2.3. Plasmid Construction

*NTRK2* overexpression (pcDNA3.1-*NTRK2*), *NTRK2* knockdown (three small interfering RNAs (siRNAs)), and the respective negative controls (pcDNA3.1-NC, siRNA-NC) were synthesized by GenePharma (Shanghai, China); the sequences are shown in Table 1.

### 2.4. Cell Transfection

Sheep granulosa cells were seeded at a density of 1 × 10^6^ cells per well in 6-well plates (three replicates per group) with 2 mL of complete DMEM (Gibco, Grand Island, NE, USA) per well and cultured at 37 °C under 5% saturated humidity for 10 h. When the cells reached 70–80% confluence, the original culture medium was discarded, and the cells were washed twice with 1 mL of PBS. The cells in each well of the 6-well plate were transfected with 100 pmol of siRNA or 4 μg *pcDNA3.1-NTRK2* according to the manufacturer’s protocol for Lipofectamine 2000 (Invitrogen, Carlsbad, CA, USA). After 48 h of incubation, cells and their supernatants were collected for subsequent experiments.

### 2.5. RNA Extraction and RT-qPCR

The granulosa cells were collected 48 h after transfection, and the Total RNA of granulosa cells was extracted according to the instructions of a Tiangen Tissue/Cell RNA Extraction Kit (Tiangen Biotechnology Co., Ltd., Beijing, China) and reverse-transcribed according to the instructions of a TaKaRa reverse transcription kit (Takara, Dalian, China). RT–qPCR was performed using cDNA as a template. The RT–qPCR system contained 1.6 μL of primers at a concentration of 10 μmol/μL, 10 μL of SYBR Green qPCR (Takara, Dalian, China), 2 μL of 50 ng/μL cDNA, and 6.4 μL of ddH_2_O, for a total volume of 20 μL. The reaction program included pre-denaturation at 95 °C for 5 s followed by 95 °C for 5 s and 60 °C for 30 s for a total of 40 cycles. *RPL19* from sheep was used as the reference gene for calibration, and relative mRNA expression was calculated using the 2^−ΔΔCT^ method (with three replicates per group) [36]. The RT–qPCR primer sequences are shown in Table 2, and all primers used were synthesized by the Sangon company (Sangon, Shanghai, China). 

### 2.6. Protein Extraction and Western Blot Assay

The granulosa cells were collected 72 h after transfection and the proteins of sheep granulosa cells were extracted using a Total Protein Extraction Kit (Solarbio, Beijing, China). Trypsin-digested (Gibco, Grand Island, NE, USA) granulosa cells were centrifuged at 4 °C and 1500 rpm for 5 min, and the precipitate was collected and added to a protein lysate containing a protease inhibitor (Gibco, Grand Island, NE, USA). The homogenate was lysed for 30 min with shaking every 10 min. After centrifugation at 4 °C and 12,000 rpm for 30 min, the supernatant was collected. The BCA curve was drawn according to the BCA protein concentration determination kit (Solarbio, Beijing, China), and the protein concentration was calculated. SDS loading buffer (5×) (Beyotime, Shanghai, China) was added at a ratio of 1:4. After the mixture was shaken, it was heated in a metal water bath at 100 °C for 5 min, and the denatured proteins were stored at −80 °C. Samples of 30 μg of denatured protein were run on a 10% SDS–PAGE gel. The electrophoresis conditions were 80 V for 20 min and 120 V for 60 min. After electrophoresis, the activated PVDF membrane (Merck Millipore, Darmstadt, Germany) was incubated on the gel for membrane transfer. The transfer membrane condition was 150 mA for 90 min. After transfer, the membrane was blocked at room temperature for 2 h. The membrane was incubated with the recommended dilutions of anti-NTRK2, anti-GAPDH, anti-cyclin-D2, anti-CDK4, anti-PI3K, anti-P-PI3K, anti-AKT, and anti-P-AKT antibodies overnight at 4 °C. TBST (Solarbio, Beijing, China) washes were performed 5 times for 5 min each time. The membrane was incubated with the secondary antibody at room temperature for 2 h and washed with TBST 5 times. Ultrasensitive ECL chemiluminescence reagents (Beyotime, Shanghai, China) were used for color presentation, and an OdysseyCLX imaging system (Jiapeng, Shanghai, China) was used to expose and photograph the membrane and save the images. The grayscale values of the bands were analyzed using ImageJ software (Version 1.54h), and the ratio of the gray value of the target protein to that of GAPDH was calculated as the relative expression level of the target protein (three replicates per group). The antibodies used in this experiment are shown in Table 3.

### 2.7. Cell Counting Experiments

Granulosa cells transfected with pcDNA3.1-*NTRK2* (overexpression group), pcDNA3.1-*NTRK2* NC (overexpression control group), si-*NTRK2* (interference group), and si-*NTRK2* NC (interference control group) were inoculated into 96-well plates at a density of 1 × 10^4^ cells per well. The cells were incubated at 37 °C under 5% CO_2_ with saturated humidity for 6 h until granulosa cells were attached to the wall. According to the instructions of the Cell Counting Kit-8 (CCK-8) (Solarbio, Beijing, China), 10 μL of CCK-8 solution was added to each well after 0, 6, 12, 24, and 48 h of cell growth, and the cells were incubated in the incubator for 1 h. The proliferation of granulosa cells was assessed according to the absorbance at 450 nm as measured with a plate reader (Bio-Rad, iMark™, Hercules, CA, USA) (three replicates per group).

### 2.8. EdU Assay

Ovarian granulosa cells were cultured at a density of 1 × 10^6^ per well in 6-well plates fitted with cell slides. EdU working solution was prepared according to the instructions of a BeyoClick EdU-488 Cell Proliferation Detection Kit (Beyotime, Shanghai, China). Then, 500 μL (10 μmol/L) of EdU working solution was added to each well, and the cells were incubated for 2 h, after which the culture medium was removed. One milliliter of paraformaldehyde was added to fix the cells for 15 min. The fixative was removed, and the cells were washed three times with PBS for 5 min each time. The washing solution was removed, and each well was incubated with 1 mL of permeabilization solution for 10–15 min at room temperature. The permeabilization solution was removed, and the cells were washed with 1 mL of PBS 1–2 times per well for 3–5 min each time. Then, 0.5 mL of reaction solution was added to each well, the plate was gently shaken to ensure that the reaction mixture covered the samples evenly, and the cells were incubated for 30 min at room temperature while protected from light. The Click reaction solution was removed, and the cells were washed 3 times with washing solution for 3–5 min each time. One milliliter of 1× Hoechst 33342 solution (Beyotime, Shanghai, China) was added to each well for nuclear staining, and the cells were incubated for 10 min at room temperature while protected from light. The 1× Hoechst 33342 solution was removed, and the cells were washed 3 times with the washing solution for 3–5 min each time. The cell crawls were removed from the six-well plates and placed on a slide to observe and quantify the number of EdU-stained cells with fluorescence microscopy. Three fields of view were randomly selected for statistical analysis.

### 2.9. Analysis of Steroid Hormone Secretion

The supernatant of the pellet cell culture was extracted 48 h after transfection and centrifuged at 3000 rpm for 30 min. The cells precipitate was removed, and antibody–antigen–enzyme complexes were then prepared using purified sheep estradiol and progesterone from a sheep hormone ELISA kit (Meimian, Yancheng, China) and an HRP-labeled antibody. After thorough washing, the TMB substrate was added for color development. TMB was converted to a blue substance via the addition of the HRP enzyme and finally to a yellow substance under the action of acid; the intensity of the color was positively correlated with the corresponding hormone level in the sample. The absorbance (OD) was measured at 450 nm using a microplate reader, and the concentration of the corresponding sheep hormone in the sample was calculated from the standard curve (three replicates per group).

### 2.10. Construction of Protein–Protein Interaction (PPI) Network

A protein–protein interaction (PPI) network of target genes with PI3K-AKT signaling pathway was constructed by uploading the target genes with genes related to the PI3K-AKT signaling pathway into the STRING software (Version 12.0, https://cn.string-db.org/ accessed on 10 May 2023, Bio: Ovis aries). The minimum interaction score required was medium confidence (0.400).

### 2.11. Statistical Analysis

Statistical analysis was performed using SPSS 19.0 software. Three biological replicates were performed for each group in RT-qPCR, Western blot, CCK8 and EdU experiments, and the data are expressed as the mean ± standard deviation. Histograms were plotted using GraphPad Prism 9 software. * *p* < 0.05 and ** *p* < 0.01 indicate statistically significant differences between groups.

## 3. Results

### 3.1. Identification of Sheep Granulosa Cells and Localization of NTRK2 in Granulosa Cells

**Figure 1:**
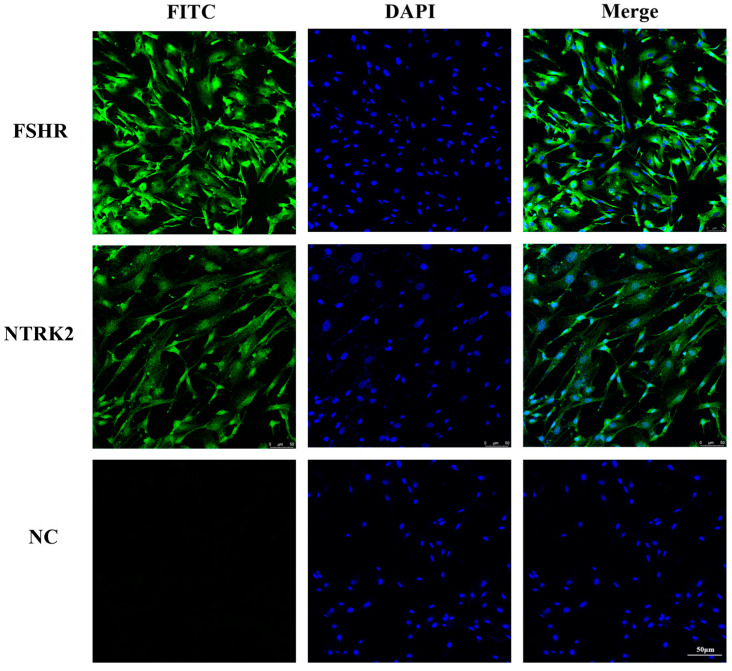
Expression localization of ovarian granulosa cell marker proteins FSHR and NTRK2 in sheep GCs. Green: FSHR/NTRK2; blue: DAPI; magnification = 200×; scale = 50 μm.

To confirm that the cells isolated and cultured in this study were sheep ovarian granulosa cells, the expression of FSHR in the cells was detected using immunofluorescence. The results showed that FSHR was abundantly expressed in the isolated cells, indicating that these cells were granulosa cells. The expression location of NTRK2 in the granulosa cells was also detected using immunofluorescence, and the results showed that the NTRK2 was abundantly expressed in sheep granulosa cells (Figure 1).

### 3.2. NTRK2 Promotes the Proliferation of Sheep Granulosa Cells

**Figure 2:**
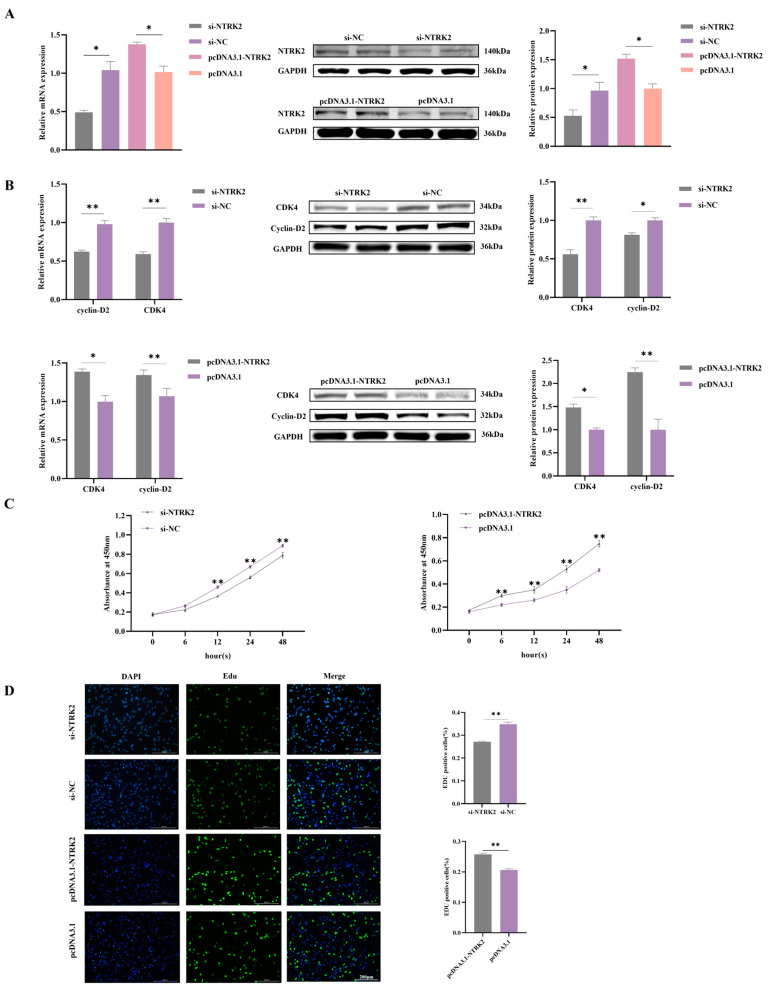
Effect of *NTRK2* on the proliferation of GCs in sheep. (**A**) Transcriptional and protein-level validation of *NTRK2* interference and overexpression efficiency in granulosa cells. (**B**) Detection of cell cycle protein-D2 and *CDK4* mRNA expression levels in sheep GCs after overexpression or inhibition of *NTRK2* and the results of protein expression levels and gray value analysis. (**C**) CCK-8 assay for the proliferation of sheep GCs after overexpression or inhibition of *NTRK2.* (**D**) EdU assay for the proliferation of sheep GCs after overexpression or inhibition of *NTRK2*. magnification = 200×; scale = 200 μm; * *p* < 0.05; ** *p* < 0.01.

To investigate the function of *NTRK2* in sheep GCs, interfering RNA for *NTRK2* (si-*NTRK2* and si-*NTRK2* NC) and an overexpression vector (pcDNA3.1-*NTRK2* and pcDNA3.1-*NTRK2* NC) constructed in vitro were transfected into GCs to detect the effect of *NTRK2* on GC proliferation. The transcriptional and protein-level validation of *NTRK2* interference and overexpression efficiency in granulosa cells was determined (Figure 2A). The results of RT–qPCR showed that the expression of the cells proliferation markers *CDK4* and *cyclin-D2* in sheep granulosa cells was significantly higher in the *NTRK2* overexpression group than in the NC group, and the opposite was true after the inhibition of *NTRK2* expression (*p* < 0.05) (Figure 2B). The protein levels were consistent with the mRNA levels (Figure 2B). The results of the CCK-8 experiments showed that the overexpression of *NTRK2* significantly promoted the proliferation of granulosa cells, whereas the inhibition of *NTRK2* expression suppressed the proliferation of ovarian granulosa cells (Figure 2C). The results of the EdU experiments also demonstrated that the overexpression of *NTRK2* significantly promoted the proliferation of granulosa cells, whereas the inhibition of *NTRK2* expression showed the opposite tendency (Figure 2C,D). These results suggested that *NTRK2* promoted the proliferation of sheep granulosa cells.

### 3.3. NTRK2 Promotes Estrogen and Progesterone Secretion in Sheep Granulosa Cells

**Figure 3:**
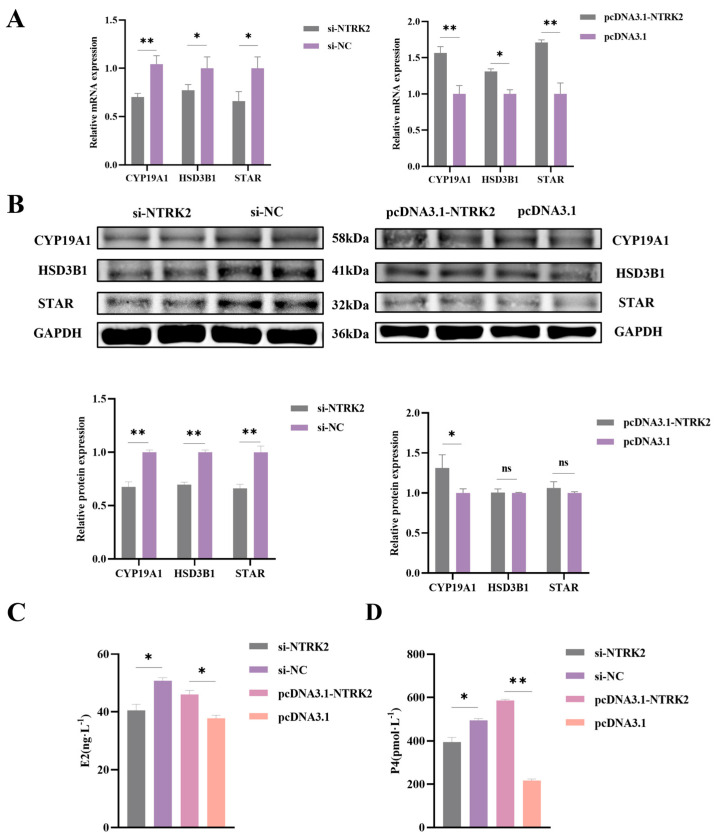
Effects of *NTRK2* overexpression or inhibition on steroid hormone secretion by sheep granulosa cells. (**A**) The expression of steroid secretion-related genes in sheep granulosa cells after *NTRK2* overexpression or inhibition. (**B**) The protein levels of genes related to steroid secretion in sheep granulosa cells after *NTRK2* overexpression or inhibition. (**C**) The level of E_2_ secretion in sheep granulosa cells after *NTRK2* overexpression or inhibition. (**D**) The level of P_4_ secretion in sheep granulosa cells after *NTRK2* overexpression or inhibition. * *p* < 0.05, ** *p* < 0.01, ns, no significance.

To determine the effect of *NTRK2* on granulosa cells, the RT-qPCR and Western blot assay were used to evaluate the expressions of the steroid hormone-secretion-related genes cytochrome P450 family 19 subfamily A member 1 (*CYP19A1*), steroidogenic acute regulatory protein (*STAR*), and hydroxy-δ-5-steroid dehydrogenase (*HSD3B1*) in sheep granulosa cells. The results of the RT-qPCR showed that the overexpression of *NTRK2* significantly increased the expression of *CYP19A1* and *STAR* mRNA (*p* < 0.01) and significantly increased the expression of *HSD3B1* mRNA (*p* < 0.05). However, inhibition of *NTRK2* significantly decreased the expression of *CYP19A1* mRNA (*p* < 0.01) and the expression of *STAR* and *HSD3B1* mRNA (*p* < 0.05) (Figure 3A). Western blot results showed that overexpression of *NTRK2* significantly increased the expression of CYP19A1 (*p* < 0.01) but had no significant effect on the expression of HSD3B1 and STAR (*p* > 0.05). Inhibition of *NTRK2* significantly decreased the expression of CYP19A1, STAR, and HSD3B1 proteins (*p* > 0.05) (Figure 3B). In addition, after overexpression of *NTRK2*, the secretion level of E2 and P4 in granulosa cells was significantly increased (*p* < 0.05), and the secretion level of P4 was extremely significantly increased (*p* < 0.01), while after inhibition of *NTRK2*, the secretion levels of E2 and P4 in granulosa cells were significantly decreased (*p* < 0.05) (Figure 3C,D). As a key enzyme in the steroid synthesis pathway in the ovary, CYP19A1 converts testosterone in the ovaries into estradiol, and testosterone also synthesizes progesterone through a series of metabolic steps. These results suggest that *NTRK2* in sheep granulosa cells promotes E_2_ and P_4_ secretion by facilitating the expression of steroid-secretion-related genes such as *CYP19A1*.

### 3.4. NTRK2 Activates the PI3K/AKT Signaling Pathway in Sheep Granulosa Cells

**Figure 4:**
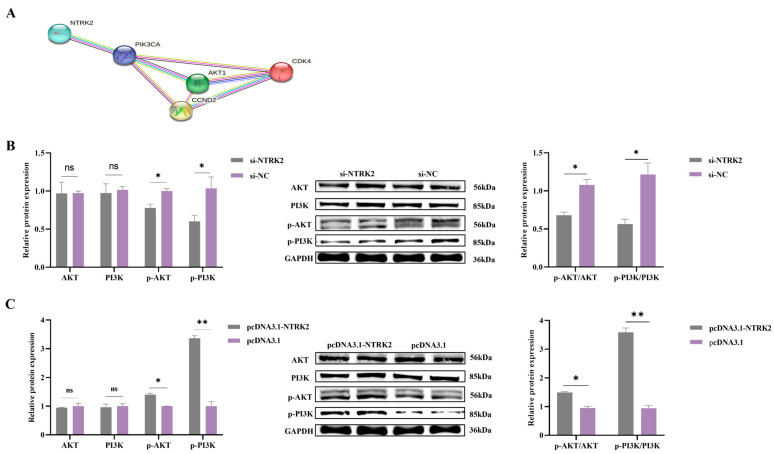
PI3K and AKT protein levels and protein phosphorylation levels were detected. (**A**) Results of PPI analysis. (**B**) PI3K and AKT protein levels and protein phosphorylation levels after inhibition of *NTRK2* expression. (**C**) PI3K and AKT protein levels and protein phosphorylation levels after overexpression of *NTRK2*. * p < 0.05; ** *p* < 0.01, ^ns^ *p* > 0.05.

Previous studies have shown that *NTRK2* is involved in the PI3K/Akt pathway, which plays a key role in cell growth, proliferation, apoptosis, and other biological processes, prompting us to study the potential effects of *NTRK2* on key genes in this signaling cascade [37]. PPI protein interaction analysis confirmed that NTRK2 interacts with PI3K and AKT (Figure 4A). Western blotting results showed that overexpression or inhibition of NTRK2 had no significant effects on the levels of PI3K and AKT (*p* > 0.05). However, after overexpression of NTRK2, the phosphorylation levels of PI3K and AKT protein in sheep GCs were significantly increased, while the opposite was true after inhibition of NTRK2 expression (*p* < 0.05) (Figure 4B,C). These results suggest that NTRK2 can affect the proliferation of sheep granulosa cells by activating the PI3K/AKT signaling pathway by regulating its phosphorylation level.

## 4. Discussion

The sheep industry is an indispensable part of the livestock industry, so improving sheep fertility has become increasingly important. A series of physiological processes in the ovaries contribute to the reproduction process of sheep [38]. The follicle is the most basic structural and functional unit of the ovaries in females, and its physiological states, including recruitment [39], selection [40], innervation [41], maturation, and ovulation [42], strongly affect the reproductive performance of sheep. In recent years, several studies have identified a variety of neurotrophic factors [30] and receptors commonly present in the mammalian reproductive system that affect the growth [43] and differentiation of somatic and germ cells in the gonads in an autocrine and paracrine manner [44], regulating reproductive organ development and reproductive function [45]. Neurotrophic factors have been shown to have effects on sex determination, fertilization, and pregnancy. In vitro addition of the neurotrophic factor *BDNF* and its receptor *NTRK2* has both direct and indirect effects on early embryonic development and granulosa cell growth in cattle [25]. In buffalo, *BDNF* promotes oocyte maturation and early embryonic development by binding to *NTRK2* [46]. The expression of the neurotrophic factor *BDNF* and its receptor *NTRK2* also varies in the uterus and oviducts of ewes at different periods of pregnancy, suggesting that these molecules have regulatory effects on gestational activity [47]. In a previous study, the brain-derived neurotrophic factor receptor was shown to influence ovarian function [17]. *NTRK2* is significantly expressed in the ovaries of infant mice, and *NTRK2*-deficient mice fail to maintain follicle growth and exhibit significant damage to follicular tissue and substantial oocyte death. *NTRK2* mediates the supportive effects of neurotrophic factors 4/5 and brain-derived neurotrophic factor in early ovarian follicle development. A deficiency of *NTRK2* receptors reduces granulosa cell (GC) proliferation and delays follicle growth [48]. 

The *NTRK2* gene has been shown to promote proliferation in a variety of cells. For example, in ovarian cancer cells, the interaction between *EGFR* and *NTRK2* enhances the migration and proliferation of ovarian cancer cells by increasing the phosphorylation of AKT [49]. In endometrial cells, *BDNF* promoted endometrial cell proliferation through the TrkB-ERK1/2 signaling pathway [50]. Moreover, in mice, BDNF/TrkB signaling also enhanced the proliferation of smooth muscle cells in mice with pulmonary hypertension [51]. However, in sheep ovarian granulosa cells, the function of the *NTRK2* gene has rarely been reported, which provides a direction for us to investigate the role of *NTRK2* in sheep ovarian granulosa cells in depth. Granulosa cells (GCs) form the largest cell population in the follicle, and the physiological functions of GCs, including estrogen synthesis and secretion, proliferation and apoptosis, and nutrient exchange, are essential for the normal development of oocytes and follicles [52]. During follicle maturation, the expression of *cyclin-D2* and *CDK4* in GCs promotes oocyte G1 division [53], which in turn affects oocyte maturation. In mouse studies, it has been found that inhibiting the expression of *Cyclin-D2* and *CDK4* in mouse ovaries significantly inhibits follicle development and GC proliferation [54]. The secretion of E_2_ and P_4_ by GCs regulates the entire process from follicle development to ovulation, and the secretion levels of these hormones are proportional to the number of ovulations, and vice versa [55,56]. It has been shown that *BDNF* binds to *NTRK2* receptors mainly in GCs and plays an important role in ovarian ovulation [45]. The *NTRK2* signaling pathway plays important roles in follicle growth and steroidogenesis in some animals, and the binding of *NTRK2* to its ligands affects steroid hormone synthesis [25]. In this study, utilizing cell counting and EdU analysis, we observed a significant enhancement in the growth of granulosa cells due to the overexpression of *NTRK2*. Furthermore, there was a marked increase in the expression levels of genes associated with cell proliferation, such as *cyclin-D2* and *CDK4*, in sheep GCs, thereby further facilitating the process of ovarian granulosa cell proliferation. This discovery aligns with previous research on other forms of cell proliferation. Additionally, overexpression of *NTRK2* correlated with elevated secretion of estrogen (E_2_) and progesterone (P_4_) in sheep GCs. Conversely, inhibition of *NTRK2* expression had the opposite effect. Collectively, these findings substantiate the pivotal role played by *NTRK2* in sheep ovarian granulosa cells; not only does it promote cell proliferation, but it also regulates hormone secretion.

Many RTK family genes promote granulosa cell proliferation during follicular development by mediating the PI3K/AKT signaling pathway. It has been shown that ovarian functions and pathologies are regulated by phosphatidylinositol-3 kinase (PI3K)/AKT signaling, including primordial follicle recruitment, granulosa cell proliferation, polycystic ovary syndrome (PCOS), and premature ovarian failure (POF) [57]. Upon the binding of BDNF to *NTRK2,* the Trk receptor stimulates the PI3K heterodimer, leading to activation of the kinases *PDK-1* and *AKT* [58]. Subsequently, *AKT* further activates transcription factors such as *FRK* (forkhead family transcription factor), *BAD*, and *GSK-3*, which affect GC proliferation [58]. The level of *AKT* protein phosphorylation increases following the activation of the PI3K-AKT signaling pathway, which in turn promotes granulosa cell proliferation [59]. To verify the role of *NTRK2* in the PI3K-AKT signaling pathway, the protein levels of PI3K and AKT as well as changes in their phosphorylation levels were evaluated in this study. The results suggest that *NTRK2* promoted the proliferation of GCs by regulating changes in the expression of *CDK4* and *cyclin-D2* mRNA as well as changes in the phosphorylation levels of the PI3K and AKT proteins. 

## 5. Conclusions

In summary, our in vitro transfection study confirmed the significant role of *NTRK2* in promoting the proliferation of sheep granulosa cells, altering reproductive hormone secretion, and participating in the PI3K/AKT signaling pathway. These findings provide new theoretical support for studying the growth and development of sheep granulosa cells. Although there is growing evidence supporting *NTRK2* as a key gene for follicular development in sheep, further in vitro and in vivo studies are needed to elucidate the specific mechanisms of action of *NTRK2* in follicular development.

## Figures and Tables

**Table 1:** Information on NTRK2 siRNA.

siRNA	Sequences (5′-3′)
siRNA-1	F: GCUGGCUGAUUGUGGGCUUTT R: AAGCCCACAAUCAGCCAGCTT
siRNA-2	F: GGAGUCUACCAGUGCUGAUTT R: AUCAGCACUGGUAGACUCCTT
siRNA-3	F: GGAUUUGUACUGUCUGGAUTT R: AUCCAGACAGUACAAAUCCTT
Negative control (NC)	F: UUCUCCGAACGUGUCACGUTT R: ACGUGACACGUUCGGAGAATT

**Table 2:** Primer information for RT–qPCR in this study.

Genes	Accession	Sequences (5′–3′)	Product Length (bp)
*NTRK2*	XM_042243072.1	F: GTTTGGCATGAAAGGCCCAG R: GAGATGTGATGCAGTGGGCT	72
*PI3K*	XM_042234194.1	F: GTGTGGGACTTATTGAGGTGGTGAG R: TGATGGAGTGTGTGGCTGTTGAAC	106
*AKT*	NM_001161857.1	F: GCAGCATCGTGTGGCAGGAC R: GTCTTGGTCAGGTGGCGTAATGG	140
*CCND2*	NM_001127290.1	F: ATCTCCTGGCAAAGATCACCAACAC R: TGTTCAGCAGCACCACCTCAATC	81
*CDK4*	XM_012158548.4	F: GCTGCTGCTGGAGATGCTGAC R: CTCTGCGTCACCTTCTGCCTTG	100
*CYP19A1*	XM_042251921.1	F: AGTCTGGGTATGTGGAGAGGAAAC R: CTGCCAAATCGGGATATGTAGTGAC	96
*HSD3B1*	XM_042255821.1	F: TGGGTGGGCTCTGAAGAATGG R: TAATTGGTCAGGATGCCGTTGTTC	135
*STAR*	NM_001009243.1	F: AGTCTGGGTATGTGGAGAGGAAAC R: CTGCCAAATCGGGATATGTAGTGAC	70
*RPL19*	XM_027974613.2	F: ATCGCCAATGCCAACTC R: CCTTTCGCTTACCTATACC	154

**Table 3:** Information on the antibodies used in this study.

Article Number	Antibodies	Dilution Ratio	Place of Origin
29961-1-AP	anti-NTRK2	1:1000	Proteintech, Wuhan, China
60225-1-Ig	anti-PI3K	1:10,000	Proteintech, Wuhan, China
60004-1-Ig	anti-GAPDH	1:100,000	Proteintech, Wuhan, China
10934-1-AP	anti-cyclin-D2	1:1000	Proteintech, Wuhan, China
11026-1-AP	anti-CDK4	1:1000	Proteintech, Wuhan, China
10176-2-AP	anti-AKT	1:2000	Proteintech, Wuhan, China
22665-1-AP	anti-FSHR	1:1000	Proteintech, Wuhan, China
ab182651	anti-P-PI3K	1:1000	Abcam, Shanghai, China
ab81283	anti-P-AKT	1:1000	Abcam, Shanghai, China
bs-1292R	anti-Aromatase	1:1000	Bioss, Beijing, China
bs-3906R	anti-HSD3B1	1:1000	Bioss, Beijing, China
bs-20387R	anti-STAR	1:1000	Bioss, Beijing, China

## Data Availability

None of the data were deposited in an official repository. The data that support the study findings are available from the authors upon request.
